# Crystal structure of 4-amino-3-(thio­phen-3-ylmeth­yl)-1*H*-1,2,4-triazole-5(4*H*)-thione

**DOI:** 10.1107/S2056989017012191

**Published:** 2017-08-30

**Authors:** Trung Vu Quoc, Linh Nguyen Ngoc, Vy Do Truc, Linh Duong Khanh, Hung Ha Manh, Chien Thang Pham, Luc Van Meervelt

**Affiliations:** a Faculty of Chemistry, Hanoi National University of Education, 136 Xuan Thuy, Cau Giay, Hanoi, Vietnam; b Faculty of Basic Sciences, University of Mining and Geology, Duc Thang, Bac Tu Liem, Hanoi, Vietnam; c VNU University of Science, Department of Inorganic Chemistry, 19 Le Thanh Tong Street, Hoan Kiem Discrict, Hanoi, Vietnam; d Department of Chemistry, KU Leuven, Biomolecular Architecture, Celestijnenlaan 200F, Leuven (Heverlee), B-3001, Belgium

**Keywords:** crystal structure, thio­phene, polythio­phene, 1,2,4-triazole-3-thione, disorder

## Abstract

In the title compound, C_7_H_8_N_4_S_2_, the thio­phene ring shows rotational disorder over two orientations in a 0.6957 (15):0.3043 (15) ratio. The plane of the 1,2,4-triazole ring makes a dihedral angle of 75.02 (17)° with the major-disorder component of the thiophene ring. In the crystal, two types of inversion dimers, described by the graph-set motifs *R*_2_^2^(8) and *R*_2_^2^(10), are formed by N—H⋯S inter­actions. Chains of mol­ecules running in the [101] direction are linked by weaker N—H⋯N inter­actions. The thio­phene ring is involved in π–π and C—H⋯π inter­actions.

## Abstract 2

The synthesis and crystal structure of a new thio­phene monomer containing an additional 1,2,4-triazole ring are reported. The compound has a V-shaped conformation with the thio­phene ring disordered over two positions by a rotation of approximately 180°.

## Chemical context   

Recently, the synthesis, characterization and anti­fungal activities, together with crystal structure determinations, of thio­phene-based heterocyclic chalcones have been investigated (Ming *et al.*, 2017 ▸). Thio­phene-containing β-diketonate complexes of copper(II) have been studied and their deposits obtained by electropolymerization have been characterized (Oyarce *et al.*, 2017 ▸). Combinations of the thio­phene ring with other heterocyclic rings have also been investigated, such as a β-keto–enol group embedded with thio­phene and pyridine moieties giving inter­esting applications in the field of solid-phase extraction (Radi *et al.*, 2016 ▸).
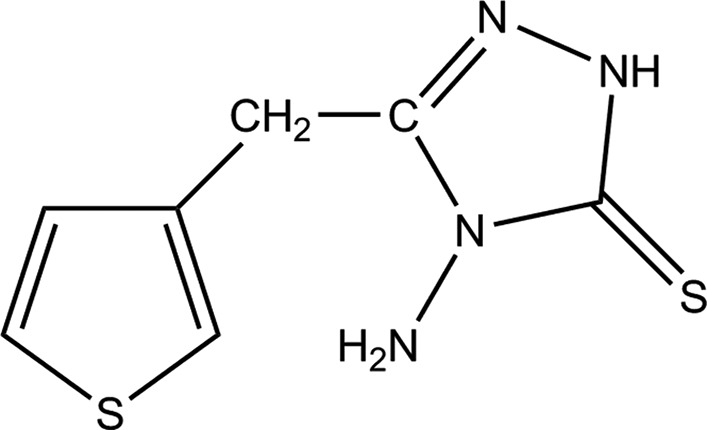


As part of our ongoing studies of new polythio­phenes and their properties (Nguyen *et al.*, 2016 ▸; Vu *et al.*, 2016 ▸; Vu Quoc *et al.*, 2017 ▸), we have synthesized a new thio­phene monomer containing an additional 1,2,4-triazole ring. The polymer obtained from 4-amino-3-(thio­phen-3-ylmeth­yl)-1*H*-1,2,4-triazole-5(4*H*)-thione using FeCl_3_ as oxidant was further characterized by IR and NMR spectroscopy, and TGA and is soluble in most common organic solvents, such as DMF and DMSO. We present here the synthesis and crystal structure of the title compound, **3**.

## Structural commentary   

**Figure 1:**
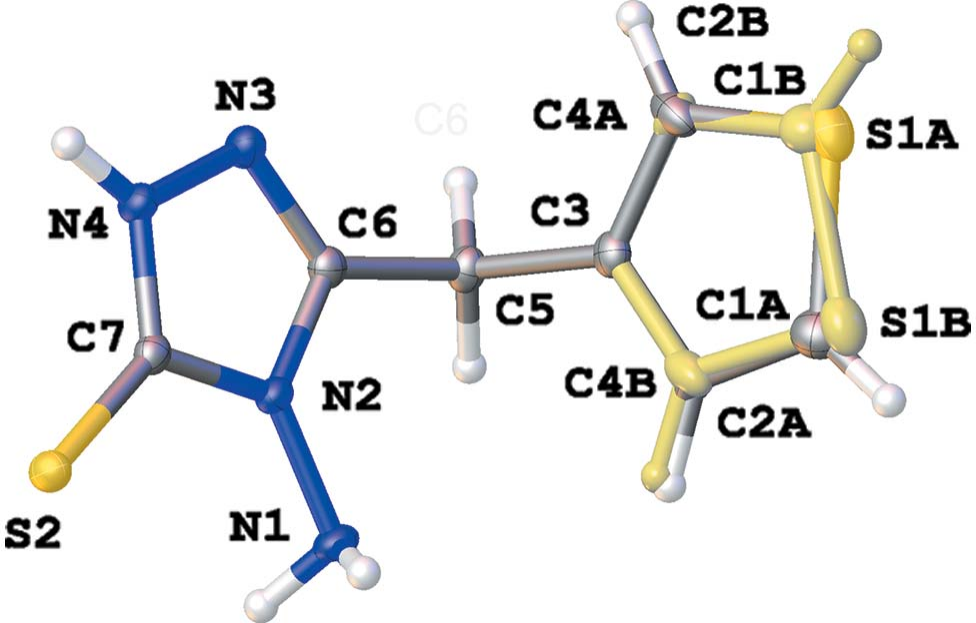
A view of the asymmetric unit of the title compound, showing the atom-labelling scheme. Displacement ellipsoids are drawn at the 50% probability level. H atoms are shown as small circles of arbitrary radii. The minor component of the disordered thio­phene rings is shown in pale yellow.

The title compound (Fig. 1 ▸) crystallizes in the monoclinic space group *P*2_1_/*n* with one mol­ecule in the asymmetric unit. The thio­phene ring is disordered over two orientations in a rotation of approximately 180° around the C5—C3 bond [occupancy factors = 0.6957 (15) for ring *A* or S1*A*/C1*A*/C2*A*/C3/C4*A* and 0.3043 (15) for ring *B* or S1*B*/C1*B*/C2*B*/C3/C4*B*]. The 1,2,4-triazole ring is almost planar (r.m.s. deviation = 0.001 Å for ring N2/N3/N4/C6/C7), with the substituents N1, S2 and C5 deviating by −0.034 (1), 0.008 (1) and 0.093 (1) Å, respectively. Due to the *sp* ^3^ character of the linking atom C5, the planes of the five-membered rings make dihedral angles of 75.02 (17) (ring *A*) and 76.4 (4)° (ring *B*), which results in a V-shaped conformation. Atom N1 clearly has an *sp* ^3^ hybridization as shown by the bond angles.

## Supra­molecular features   

**Figure 2:**
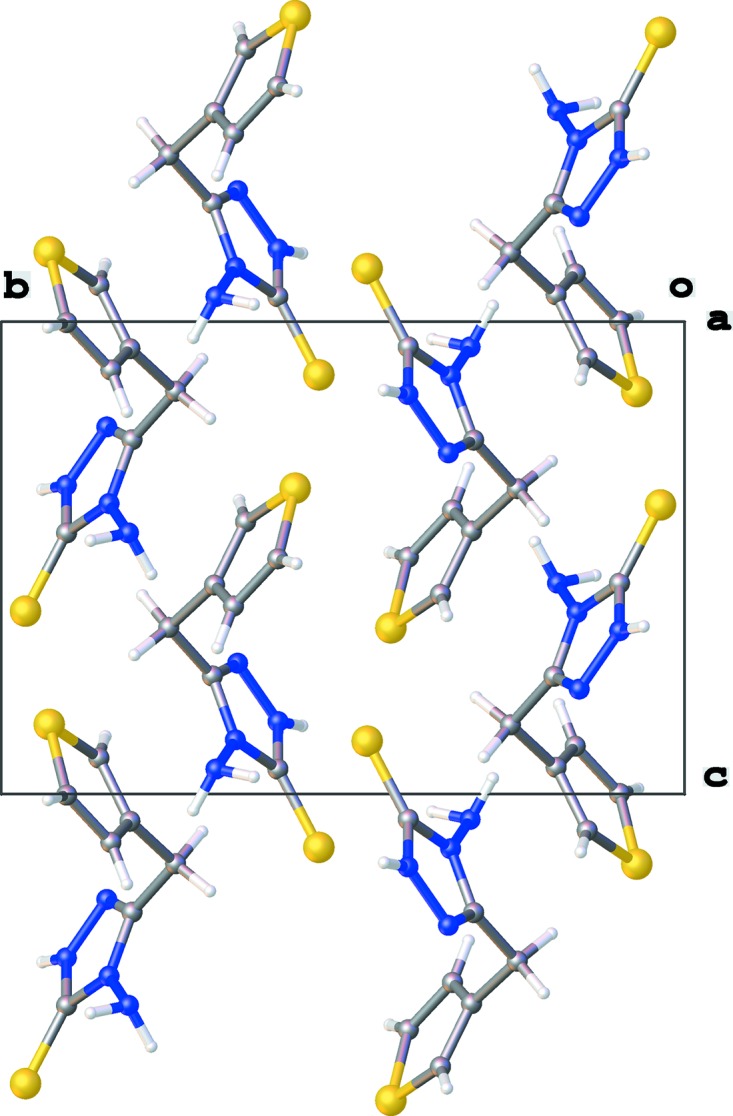
Crystal packing of the title compound shown in projection down the *a* axis.

**Figure 3:**
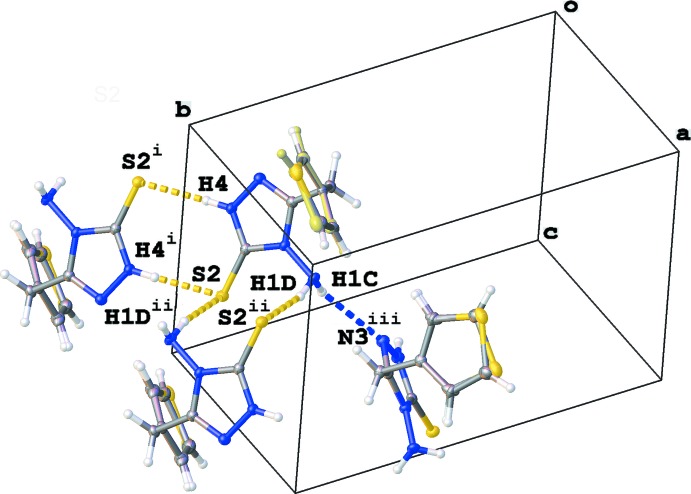
Part of the crystal packing of the title compound, showing the rings of graph-set motif 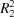
(8) and 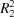
(10) formed by N—H⋯S hydrogen-bond inter­actions [see Table 1 ▸; symmetry codes: (i) −*x*, −*y* + 2, −*z* + 1; (ii) −*x* + 1, −*y* + 2, −*z* + 1; (iii) *x* + 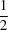, −*y* + 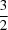, *z* + 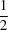] and a chain of graph-set motif *C*(5).

The crystal packing of the title compound is shown in Fig. 2 ▸. The 1*H*-1,2,4-triazole-5(4*H*)-thione ring possesses an NH_2_ group, which, in principle, can act as a donor or acceptor for hydrogen bonding, an NH group, which can act as a donor, and an N atom and C=S group, which can only act as acceptors. Two types of inversion dimers are formed (Fig. 3 ▸ and Table 1 ▸). The first one, described as graph-set motif 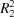
(8), involves hydrogen bonds between the NH and C=S groups, whereas in the second one, the NH_2_ group interacts with the C=S grouping, resulting in a ring structure of graph-set 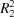
(10). The second H atom of the NH_2_ group inter­acts with the N atom of a neighbouring 1*H*-1,2,4-triazole-5(4*H*)-thione ring, resulting in chains of graph-set *C*(5) in the [101] direction (Fig. 3 ▸ and Table 1 ▸).

**Figure 4:**
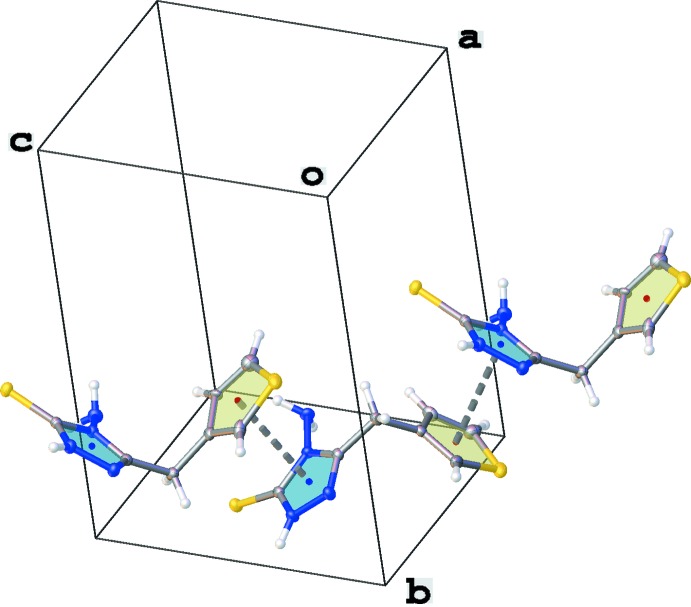
Part of the crystal packing of the title compound, showing the π–π stacking inter­actions between the thio­phene (yellow) and 1,2,4-triazole (blue) rings (only the major component of the disordered thio­phene ring is shown).

The disordered thio­phene ring is only involved in a π–π stacking inter­action with the 1,2,4-triazole ring [*Cg*1⋯*Cg*3^i^ = 3.415 (2) Å and *Cg*2⋯*Cg*3^i^ = 3.440 (5) Å; *Cg*1, *Cg*2 and *Cg*3 are the centroids of ring *A*, ring *B* and the 1,2,4-triazole ring, respectively; symmetry code: (i) *x* + 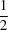, −*y* + 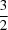, *z* − 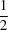; Fig. 4 ▸]. The crystal packing shows a weak C—H⋯π inter­action (Table 1 ▸) and contains no voids.

**Figure 5:**
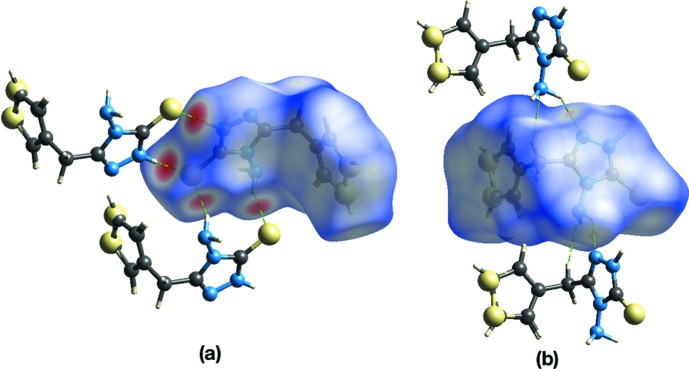
Hirshfeld surface for title compound mapped over *d*_norm_ over the range −0.436 to 1.179 a.u., highlighting (*a*) the N—H⋯S hydrogen bonding and (*b*) the N—H⋯N and C—H⋯N inter­actions.

The packing was further investigated by an analysis of the Hirshfeld surface and two-dimensional fingerprint plots using *CrystalExplorer* (McKinnon *et al.*, 2007 ▸; Spackman & Jayatilaka, 2009 ▸). The donors and acceptors corresponding to the N—H⋯S inter­actions are visible as bright-red spots in Fig. 5 ▸(*a*). The pale-red spots in Fig. 5 ▸(*b*) are the weaker N—H⋯N and C—H⋯N inter­actions. The relative contributions of the different inter­molecular inter­actions to the Hirshfeld surface area in descending order are: H⋯H (40.4%), S⋯H (26.7%), N⋯H (13.3%), C⋯H (8.2%), C⋯C (4.1%), C⋯N (3.7%), S⋯C (2.3%) and S⋯N (1.2%). This illustrates that the weak N—H⋯N and C—H⋯N inter­actions contribute significantly to the packing of the title compound.

## Database survey   

A search of the Cambridge Structural Database (CSD, Version 5.38, last update May 2017; Groom *et al.*, 2016 ▸) for structures containing an 4-amino-3-methyl-1*H*-1,2,4-triazole-5(4*H*)-thione moiety gave 69 hits; in 41 of these structures, the C=S and/or NH_2_ groups are complexed with a metal ion. The 1,2,4-triazole ring is almost planar, with the largest deviation from the best plane through the ring atoms being 0.034 Å [for the complex *mer*-tri­chlorido­(dimethyl sulfoxide-κ*S*)(4-amino-3-ethyl-1,2,4-Δ^2^-triazoline-5-thione-κ^2^ *N*,*S*)ruthenium(III) hemi­hydrate; CSD refcode KESQOO; Cingi *et al.*, 2000 ▸].

## Synthesis and crystallization   

**Figure 6:**
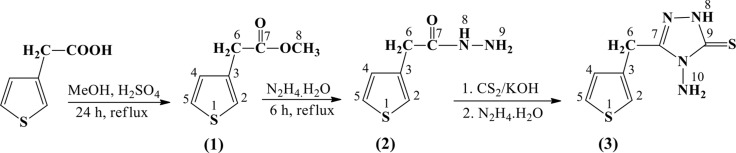
Reaction scheme for the title compound.

The reaction scheme used to synthesize the title compound, **3**, is given in Fig. 6 ▸. Methyl 2-(thio­phen-3-yl)acetate, **1**, and 2-(thio­phen-3-yl)acetohydrazide, **2**, were synthesized as described in a previous study (Vu Quoc *et al.*, 2017 ▸).

For the synthesis of 4-amino-3-(thio­phen-3-ylmeth­yl)-1*H*-1,2,4-triazole-5(4*H*)-thione, **3**, a mixture of hydrazide **2** (5 mmol), KOH (0.01 mol), ethanol (10 ml) and carbon di­sulfide (10 mmol) was stirred at room temperature until the formation of hydrogen sulfide stopped. An excess of alcohol was removed by distillation and the solid was washed with diethyl ether. A mixture of the resulting solid in water (10 ml) and hydrazine hydrate (15 ml) was then refluxed for 8 h at 353 K. The reaction mixture was cooled and neutralized with dilute hydro­chloric acid. The solid which precipitated was filtered off, washed thoroughly with water, dried and recrystallized from an ethanol–water solvent mixture (4:1 *v*/*v*) to give 0.63 g (yield 60.0%) of **3** in the form of colourless crystals (m.p. 378 K). IR (Nicolet Impact 410 FT–IR, KBr, cm^−1^): 3452 (ν_NH_), 3088, 2911 (ν_CH_), 1576 (ν_C=C_ thio­phene), 1278, 1207 (ν_C=S_). ^1^H NMR [Bruker XL-500, 500 MHz, *d*_6_-DMSO, δ (ppm), *J* (Hz)]: 7.33 (*m*, 1H, ^4^ *J* = 1.0, H^2^), 7.06 (*m*, 1H, ^2^ *J* = 1.0, ^5^ *J* = 5.0, H^4^), 7.49 (*dd*, 1H, ^2^ *J* = 3.0, ^4^ *J* = 5.0, H^5^), 4.04 (*s*, 2H, H^6^), 13.54 (s, 1H, H^8^), 5.58 (*s*, 2H, H^10^). ^13^C NMR [Bruker XL-500, 125 MHz, *d*_6_-DMSO, δ (ppm)]: 123.03 (C2), 135.61 (C3), 128.98 (C4), 126.67 (C5), 25.60 (C6), 151.55 (C7), 166.47 (C9). Calculation for C_7_H_8_N_4_S_2_: *M* = 212 a.u.

## Refinement   

Crystal data, data collection and structure refinement details are summarized in Table 2 ▸. Both thio­phene rings are disordered over two orientations by a rotation of approximately 180° around the C5—C3 bond. The final occupancy factors are 0.6957 (15) and 0.3043 (15). For the disordered thio­phene ring, bond lengths and angles were restrained to the target mean values observed in 3-CH_2_-thio­phene fragments in the CSD (Groom *et al.*, 2016 ▸) and the same anisotropic displacement parameters were used for equivalent atoms. The H atoms attached to atoms N1 and N4 were found in a difference density Fourier map and refined freely. The other H atoms were placed at calculated positions and refined in riding mode, with C—H distances of 0.95 (aromatic) and 0.99 Å (CH_2_), and isotropic displacement parameters equal to 1.2*U*_eq_ of the parent atoms. In the final cycles of refinement, two reflections showing very poor agreement were omitted as outliers.

## Supplementary Material

Crystal structure: contains datablock(s) I. DOI: 10.1107/S2056989017012191/zp2023sup1.cif

Structure factors: contains datablock(s) I. DOI: 10.1107/S2056989017012191/zp2023Isup2.hkl

Supporting information file. DOI: 10.1107/S2056989017012191/zp2023Isup3.cml

CCDC reference: 1570281

Additional supporting information:  crystallographic information; 3D view; checkCIF report

## Figures and Tables

**Table 1:** Hydrogen-bond geometry (Å, °) *Cg*3 is the centroid of the N2/N3/N4/C6/C7 ring.

*D*—H⋯*A*	*D*—H	H⋯*A*	*D*⋯*A*	*D*—H⋯*A*
N1—H1*C*⋯N3^i^	0.909 (15)	2.622 (15)	3.3847 (13)	142.0 (12)
N1—H1*D*⋯S2^ii^	0.849 (17)	2.628 (16)	3.4163 (9)	154.9 (13)
N4—H4⋯S2^iii^	0.890 (16)	2.395 (15)	3.2847 (9)	178.2 (12)
C1*B*—H1*B*⋯*Cg*3^iv^	0.95	2.78	3.503 (11)	134

Symmetry codes: (i) 
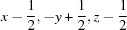
; (ii) 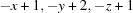; (iii) 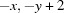, 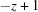; (iv) 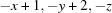.

**Table 2:** Experimental details

Crystal data	
Chemical formula	C_7_H_8_N_4_S_2_
*M*_r_	212.29
Crystal system, space group	Monoclinic, *P*2_1_/*n*
Temperature (K)	100
*a*, *b*, *c* (Å)	7.6904 (4), 13.0429 (7), 9.0220 (4)
β (°)	90.081 (2)
*V* (Å^3^)	904.95 (8)
*Z*	4
Radiation type	Mo *K*α
μ (mm^−1^)	0.54
Crystal size (mm)	0.32 × 0.20 × 0.08

Data collection	
Diffractometer	Bruker APEXII CCD
Absorption correction	Multi-scan (*SADABS*; Bruker, 2014 ▸)
*T*_min_, *T*_max_	0.710, 0.746
No. of measured, independent and observed [*I* > 2σ(*I*)] reflections	27159, 2784, 2536
*R*_int_	0.025
(sin θ/λ)_max_ (Å^−1^)	0.718

Refinement	
*R*[*F* ^2^ > 2σ(*F* ^2^)], *wR*(*F* ^2^), *S*	0.027, 0.071, 1.05
No. of reflections	2784
No. of parameters	143
No. of restraints	20
H-atom treatment	H atoms treated by a mixture of independent and constrained refinement
Δρ_max_, Δρ_min_ (e Å^−3^)	0.43, −0.35

Computer programs: *APEX2* (Bruker, 2014 ▸), *SAINT* (Bruker, 2013 ▸), *SHELXS97* (Sheldrick, 2008 ▸), *SHELXL2014* (Sheldrick, 2015 ▸) and *OLEX2* (Dolomanov *et al.*, 2009 ▸).

## supplementary crystallographic information

### Crystal data

**Table:**

C_7_H_8_N_4_S_2_	*F*(000) = 440
*M**_r_* = 212.29	*D*_x_ = 1.558 Mg m^−^^3^
Monoclinic, *P*2_1_/*n*	Mo *K*α radiation, λ = 0.71073 Å
*a* = 7.6904 (4) Å	Cell parameters from 9996 reflections
*b* = 13.0429 (7) Å	θ = 3.1–30.7°
*c* = 9.0220 (4) Å	µ = 0.54 mm^−^^1^
β = 90.081 (2)°	*T* = 100 K
*V* = 904.95 (8) Å^3^	Block, colorless
*Z* = 4	0.32 × 0.20 × 0.08 mm

### Data collection

**Table:**

Bruker APEXII CCD diffractometer	2536 reflections with *I* > 2σ(*I*)
φ and ω scans	*R*_int_ = 0.025
Absorption correction: multi-scan (SADABS; Bruker, 2014)	θ_max_ = 30.7°, θ_min_ = 2.8°
*T*_min_ = 0.710, *T*_max_ = 0.746	*h* = −11→11
27159 measured reflections	*k* = −18→18
2784 independent reflections	*l* = −12→12

### Refinement

**Table:**

Refinement on *F*^2^	20 restraints
Least-squares matrix: full	Hydrogen site location: mixed
*R*[*F*^2^ > 2σ(*F*^2^)] = 0.027	H atoms treated by a mixture of independent and constrained refinement
*wR*(*F*^2^) = 0.071	*w* = 1/[σ^2^(*F*_o_^2^) + (0.0367*P*)^2^ + 0.3687*P*] where *P* = (*F*_o_^2^ + 2*F*_c_^2^)/3
*S* = 1.05	(Δ/σ)_max_ = 0.001
2784 reflections	Δρ_max_ = 0.43 e Å^−^^3^
143 parameters	Δρ_min_ = −0.35 e Å^−^^3^

### Special details

**Table:**

Geometry. All esds (except the esd in the dihedral angle between two l.s. planes) are estimated using the full covariance matrix. The cell esds are taken into account individually in the estimation of esds in distances, angles and torsion angles; correlations between esds in cell parameters are only used when they are defined by crystal symmetry. An approximate (isotropic) treatment of cell esds is used for estimating esds involving l.s. planes.

### Fractional atomic coordinates and isotropic or equivalent isotropic displacement parameters (Å^2^)

**Table:**

	*x*	*y*	*z*	*U*_iso_*/*U*_eq_	Occ. (<1)
S1A	0.63885 (12)	0.92996 (9)	−0.14786 (11)	0.02217 (15)	0.6957 (15)
C1A	0.7802 (4)	0.9045 (2)	0.0018 (3)	0.0237 (6)	0.6957 (15)
H1A	0.894252	0.931612	0.011838	0.028*	0.6957 (15)
C2A	0.7019 (5)	0.8398 (5)	0.1017 (6)	0.0164 (5)	0.6957 (15)
H2A	0.755868	0.816279	0.190184	0.020*	0.6957 (15)
S1B	0.7968 (3)	0.92242 (15)	−0.0160 (2)	0.02217 (15)	0.3043 (15)
C1B	0.6169 (13)	0.9240 (9)	−0.1362 (11)	0.0237 (6)	0.3043 (15)
H1B	0.606846	0.962783	−0.224912	0.028*	0.3043 (15)
C2B	0.4838 (15)	0.8542 (12)	−0.0748 (15)	0.0164 (5)	0.3043 (15)
H2B	0.376467	0.840204	−0.122989	0.020*	0.3043 (15)
C3	0.52961 (13)	0.81192 (7)	0.05711 (11)	0.01448 (18)	
C4A	0.4817 (8)	0.8574 (6)	−0.0767 (6)	0.0211 (5)	0.6957 (15)
H4A	0.371471	0.847945	−0.122328	0.025*	0.6957 (15)
C4B	0.6897 (15)	0.8412 (12)	0.1007 (13)	0.0211 (5)	0.3043 (15)
H4B	0.739939	0.817943	0.190900	0.025*	0.3043 (15)
C5	0.40813 (13)	0.74501 (8)	0.14692 (11)	0.01518 (18)	
H5A	0.330663	0.706410	0.079246	0.018*	
H5B	0.476853	0.694874	0.204932	0.018*	
C6	0.30116 (12)	0.80879 (7)	0.24957 (10)	0.01347 (17)	
N3	0.14624 (11)	0.84574 (7)	0.22259 (9)	0.01560 (16)	
N4	0.10978 (11)	0.90487 (7)	0.34611 (9)	0.01478 (16)	
H4	0.011 (2)	0.9399 (12)	0.3552 (18)	0.030 (4)*	
C7	0.23720 (12)	0.90463 (7)	0.44686 (10)	0.01315 (17)	
N2	0.36064 (10)	0.84243 (6)	0.38483 (9)	0.01294 (15)	
S2	0.25221 (3)	0.96465 (2)	0.61239 (3)	0.01586 (7)	
N1	0.52086 (11)	0.81396 (7)	0.44672 (10)	0.01611 (17)	
H1C	0.5008 (19)	0.7808 (12)	0.5337 (17)	0.023 (4)*	
H1D	0.576 (2)	0.8692 (13)	0.4631 (17)	0.028 (4)*	

### Atomic displacement parameters (Å^2^)

**Table:**

	*U*^11^	*U*^22^	*U*^33^	*U*^12^	*U*^13^	*U*^23^
S1A	0.0285 (3)	0.0166 (2)	0.0214 (2)	0.0005 (2)	0.0079 (2)	0.00146 (16)
C1A	0.0256 (11)	0.0213 (13)	0.0241 (12)	0.0030 (9)	−0.0027 (9)	0.0011 (8)
C2A	0.0091 (10)	0.0171 (9)	0.0229 (9)	−0.0038 (9)	0.0006 (7)	−0.0041 (7)
S1B	0.0285 (3)	0.0166 (2)	0.0214 (2)	0.0005 (2)	0.0079 (2)	0.00146 (16)
C1B	0.0256 (11)	0.0213 (13)	0.0241 (12)	0.0030 (9)	−0.0027 (9)	0.0011 (8)
C2B	0.0091 (10)	0.0171 (9)	0.0229 (9)	−0.0038 (9)	0.0006 (7)	−0.0041 (7)
C3	0.0150 (4)	0.0134 (4)	0.0151 (4)	0.0023 (3)	0.0032 (3)	−0.0021 (3)
C4A	0.0270 (10)	0.0233 (12)	0.0131 (8)	0.0096 (8)	0.0020 (7)	0.0019 (7)
C4B	0.0270 (10)	0.0233 (12)	0.0131 (8)	0.0096 (8)	0.0020 (7)	0.0019 (7)
C5	0.0145 (4)	0.0144 (4)	0.0167 (4)	−0.0002 (3)	0.0023 (3)	−0.0024 (3)
C6	0.0131 (4)	0.0131 (4)	0.0142 (4)	−0.0012 (3)	0.0011 (3)	0.0001 (3)
N3	0.0140 (4)	0.0182 (4)	0.0146 (4)	0.0010 (3)	0.0005 (3)	−0.0022 (3)
N4	0.0124 (4)	0.0169 (4)	0.0151 (4)	0.0027 (3)	0.0001 (3)	−0.0015 (3)
C7	0.0122 (4)	0.0127 (4)	0.0145 (4)	0.0002 (3)	0.0019 (3)	0.0020 (3)
N2	0.0105 (3)	0.0146 (4)	0.0138 (3)	0.0015 (3)	0.0000 (3)	0.0007 (3)
S2	0.01442 (12)	0.01893 (13)	0.01423 (12)	0.00179 (8)	−0.00036 (8)	−0.00250 (8)
N1	0.0113 (4)	0.0190 (4)	0.0181 (4)	0.0018 (3)	−0.0029 (3)	0.0020 (3)

### Geometric parameters (Å, º)

**Table:**

S1Aa—C1A	1.763 (4)	C3—C4B	1.347 (10)
C1Aa—H1A	0.9500	C3—C5	1.5143 (14)
C1Aa—C2A	1.375 (5)	C5—H5A	0.9900
C2Aa—H2A	0.9500	C5—H5B	0.9900
S1Bb—C1B	1.757 (11)	C5—C6	1.4929 (13)
C1Bb—H1B	0.9500	C6—N3	1.3077 (13)
C1Bb—C2B	1.478 (12)	C6—N2	1.3745 (12)
C2Bb—H2B	0.9500	N3—N4	1.3842 (11)
C2Aa—C3	1.431 (3)	N4—H4	0.889 (17)
C2Bb—C3	1.357 (11)	N4—C7	1.3356 (12)
S1Aa—C4A	1.665 (5)	C7—N2	1.3689 (12)
C4Aa—H4A	0.9500	C7—S2	1.6900 (10)
S1Bb—C4B	1.707 (11)	N2—N1	1.4021 (11)
C4Bb—H4B	0.9500	N1—H1C	0.909 (15)
C3—C4A	1.394 (5)	N1—H1D	0.849 (17)
			
C2Aa—C1Aa—S1A	110.3 (3)	C3—C2Bb—H2B	123.2
C4Aa—S1Aa—C1A	92.56 (19)	C3—C5—H5B	109.5
C2Aa—C1Aa—H1A	124.8	H5A—C5—H5B	108.1
S1Aa—C1Aa—H1A	124.8	C3—C4Aa—H4A	123.7
C1Aa—C2Aa—H2A	123.9	C3—C4Bb—H4B	122.0
C2Bb—C1Bb—S1B	107.9 (7)	C6—C5—C3	110.59 (8)
C4Bb—S1Bb—C1B	90.4 (5)	C6—C5—H5A	109.5
S1Bb—C1Bb—H1B	126.1	C6—C5—H5B	109.5
C2Bb—C1Bb—H1B	126.1	N3—C6—C5	126.34 (9)
C1Bb—C2Bb—H2B	123.2	N3—C6—N2	110.46 (8)
C1Aa—C2Aa—C3	112.2 (3)	N2—C6—C5	123.07 (9)
S1Aa—C4Aa—H4A	123.7	C6—N3—N4	103.94 (8)
S1Bb—C4Bb—H4B	122.0	N3—N4—H4	122.3 (11)
C4Aa—C3—C2A	112.3 (3)	C7—N4—N3	113.41 (8)
C4Bb—C3—C2B	112.1 (6)	C7—N4—H4	124.3 (11)
C4Aa—C3—C5	123.1 (2)	N4—C7—N2	103.39 (8)
C2Aa—C3—C5	124.6 (2)	N4—C7—S2	130.50 (8)
C2Bb—C3—C5	122.9 (4)	N2—C7—S2	126.10 (7)
C4Bb—C3—C5	124.8 (4)	C6—N2—N1	124.07 (8)
C3—C5—H5A	109.5	C7—N2—C6	108.79 (8)
C3—C4Aa—S1A	112.6 (3)	C7—N2—N1	127.13 (8)
C3—C2Aa—H2A	123.9	N2—N1—H1C	108.7 (9)
C3—C4Bb—S1B	115.9 (7)	N2—N1—H1D	106.5 (11)
C3—C2Bb—C1B	113.6 (7)	H1C—N1—H1D	109.7 (14)
			
C4Aa—S1Aa—C1Aa—C2Aa	0.5 (5)	C2Aa—C3—C5—C6	89.6 (3)
S1Aa—C1Aa—C2Aa—C3	−0.2 (6)	C3—C5—C6—N3	93.21 (12)
C4Bb—S1Bb—C1Bb—C2Bb	−2.2 (12)	C3—C5—C6—N2	−82.26 (11)
S1Bb—C1Bb—C2Bb—C3	2.7 (17)	N2—C6—N3—N4	0.31 (11)
C1Bb—C2Bb—C3—C4Bb	−1.8 (18)	C5—C6—N3—N4	−175.64 (9)
C1Bb—C2Bb—C3—C5	174.8 (8)	C6—N3—N4—C7	−0.17 (11)
C1Aa—C2Aa—C3—C4Aa	−0.3 (6)	N3—N4—C7—N2	−0.04 (11)
C1Aa—C2Aa—C3—C5	−177.5 (3)	N3—N4—C7—S2	179.78 (8)
C2Aa—C3—C4Aa—S1Aa	0.7 (7)	N4—C7—N2—C6	0.23 (10)
C5—C3—C4Aa—S1Aa	178.0 (2)	S2—C7—N2—C6	−179.61 (7)
C1Aa—S1Aa—C4Aa—C3	−0.7 (5)	N4—C7—N2—N1	−178.40 (9)
C2Bb—C3—C4Bb—S1Bb	0.0 (16)	S2—C7—N2—N1	1.77 (14)
C5—C3—C4Bb—S1Bb	−176.6 (5)	N3—C6—N2—C7	−0.36 (11)
C1Bb—S1Bb—C4Bb—C3	1.4 (12)	C5—C6—N2—C7	175.75 (9)
C4Bb—C3—C5—C6	87.4 (9)	N3—C6—N2—N1	178.32 (9)
C2Bb—C3—C5—C6	−88.8 (10)	C5—C6—N2—N1	−5.57 (14)
C4Aa—C3—C5—C6	−87.3 (4)		

### Hydrogen-bond geometry (Å, º)

Cg3 is the centroid of the N2/N3/N4/C6/C7 ring.

**Table:**

*D*—H···*A*	*D*—H	H···*A*	*D*···*A*	*D*—H···*A*
N1—H1*C*···N3^i^	0.909 (15)	2.622 (15)	3.3847 (13)	142.0 (12)
N1—H1*D*···S2^ii^	0.849 (17)	2.628 (16)	3.4163 (9)	154.9 (13)
N4—H4···S2^iii^	0.890 (16)	2.395 (15)	3.2847 (9)	178.2 (12)
C1*B*—H1*B*···*Cg*3^iv^	0.95	2.78	3.503 (11)	134

Symmetry codes: (i) *x*−1/2, −*y*+1/2, *z*−1/2; (ii) −*x*+1, −*y*+2, −*z*+1; (iii) −*x*, −*y*+2, −*z*+1; (iv) −*x*+1, −*y*+2, −*z*.
